# Cerebral Processing of Prosodic Emotional Signals: Evaluation of a Network Model Using rTMS

**DOI:** 10.1371/journal.pone.0105509

**Published:** 2014-08-29

**Authors:** Heike Jacob, Carolin Brück, Christian Plewnia, Dirk Wildgruber

**Affiliations:** 1 Department of Psychiatry and Psychotherapy, University of Tübingen, Tübingen, Germany; 2 Werner Reichardt Centre for Integrative Neuroscience, University of Tübingen, Tübingen, Germany; Università di Trento, Italy

## Abstract

A great number of functional imaging studies contributed to developing a cerebral network model illustrating the processing of prosody in the brain. According to this model, the processing of prosodic emotional signals is divided into three main steps, each related to different brain areas. The present study sought to evaluate parts of the aforementioned model by using low-frequency repetitive transcranial magnetic stimulation (rTMS) over two important brain regions identified by the model: the superior temporal cortex (Experiment 1) and the inferior frontal cortex (Experiment 2). The aim of both experiments was to reduce cortical activity in the respective brain areas and evaluate whether these reductions lead to measurable behavioral effects during prosody processing. However, results obtained in this study revealed no rTMS effects on the acquired behavioral data. Possible explanations for these findings are discussed in the paper.

## Introduction

Emotions make relationships lively. There are many ways to express emotions, however, these can be summarized into two main categories: verbal (e.g. words) and nonverbal emotional signals (e.g. emotional prosody). Over the years, numerous studies have been conducted on the cerebral processing of prosodic emotional signals resulting in detailed models of the decoding process [1]–[4]. The respective models assume that the perception of prosody is based on a three-stage process including stages of extraction, integration and evaluation of acoustic information each tied to different brain structures [1]–[4]. Voice-sensitive aspects of the mid superior temporal cortex (mSTC) have been linked to the extraction of basic acoustic features [5]–[6], face-sensitive aspects of the fusiform gyrus (FFA) have been linked to the extraction of basic visual features [7], whereas posterior aspects of the superior temporal cortex (pSTC) have been implicated in the integration of prosodic and visual cues [1]–[3], [8]. Lateral frontal brain areas, particularly the inferior frontal cortices (IFC), have been suggested to contribute to the evaluation of emotional information encoded in emotional prosodic signals [9]–[11]. The model, moreover, suggests that while early stages of processing may be specific to prosody decoding, later stages involving the pSTC and IFC may constitute more general steps of decoding involved in the processing of emotional information across both the auditory and visual domain [1]–[3]. With regard to possible hemispheric differences, a recent meta-analysis of lesion studies on prosody processing revealed that although both the left and right hemisphere may contribute to prosody perception there appears to be a relative specialization of the right hemisphere for prosodic functions [12]. A second meta-analysis conducted on neuroimaging studies suggests such a rightward specialization particularly for early processing stages tied to the STC [13].

However, the described model of prosody processing [1]–[3] is mainly based on functional magnetic resonance imaging (fMRI) studies. From a methodological point of view these findings are not sufficient to draw direct conclusions about underlying causalities between brain structures and functions. Thus, the present study chose a neuromodulatory approach with repetitive transcranial magnetic stimulation (rTMS), more specifically rTMS-induced suppressions of neural activity, to evaluate the predictions of the aforementioned model [1]–[3]. By using low-frequency rTMS (<1 Hz) the stimulation-induced suppression of neural activity outlasts the train of stimulation and can be used to investigate the functional relevance of the stimulated brain area [14]. Using this approach, the present study aimed to refine the cerebral network model [1]–[3] and to disentangle necessary components from co-activated areas that are not necessarily required for the processing of prosodic emotional signals.

To our knowledge, thus far, only few studies have been published concerning rTMS effects on the processing of prosodic emotional signals. However, a recent study reported that compared to right supramarginal gyrus stimulation, disruptive high-frequency rTMS over the right temporal voice area (TVA) – an area of the STC known to be involved in the processing of human voices [5] – led to impaired detection of human voices [15]. Another study reported reaction time effects: Here, activity-decreasing low-frequency rTMS over the right superior temporal gyrus (STG) as compared to left STG or sham stimulation led to an increase of reaction times in a prosody decoding task [16]. In another study, 5 Hz rTMS over the left and right inferior frontal gyrus interfered with an emotional prosody task as indicated by longer reaction times following verum as compared to the sham stimulation [17]. Finally, another study revealed that in comparison to sham stimulation, rTMS stimulation over the right fronto-parietal operculum was accompanied by slower reaction times for the detection of withdrawal emotions (fear and sadness) expressed in prosody [18].

Referring to the model of prosody processing [1]–[3], we derived the following two hypotheses:

1. rTMS-induced reduction of the left and right STC activity should interfere with the extraction and analysis of auditory information and thus should hamper the evaluation of prosodic emotional cues whereas, given the hypothesized voice-selective nature of this brain region, no such effect should occur for visual information. Moreover, this prosody-specific impairment should be stronger for the right as compared to the left STC.
2. rTMS effects on the left and right IFC should hamper the evaluation of all kind of nonverbal emotional stimuli, independent of their respective modalities.

To test these hypotheses, two experiments were designed: Experiment 1 focused on rTMS effects following stimulation of the left and right STC, and Experiment 2 focused on rTMS effects following stimulation of the left and right IFC. To disentangle effects attributable to the disruption of activation within the target area from more general effects of stimulation, we introduced a control condition involving stimulation of a site unrelated to prosody processing. In both experiments, the posterior midline (PZ) electrode site was chosen as control region. Both experiments consisted of three blocks of offline rTMS application (one block per stimulation site), each of it followed by a computer test aiming to determine the abilities to recognize nonverbal emotional expressions in auditory, visual, and audiovisual stimuli.

## Material and Methods

### Participants

In total, 31 healthy and right-handed [19] individuals volunteered to participate in the study. The datasets of two participants had to be excluded due to technical issues. Three participants did not complete all stimulation blocks due to a feeling of discomfort and thus had to be excluded. The remaining 26 participants were divided into two experimental groups of 13 individuals each (Experiment 1: 8 females; mean age  = 27.77 years, standard deviation [*SD*]  = ±4.38 years; Experiment 2: 8 females; mean age  = 27.92 years, *SD*  = ±4.52 years). None of the participants reported any psychiatric illnesses in the Mini International Neuropsychiatric Interview [20] or neurological illnesses, nor indicated any hearing difficulties or uncorrected vision impairments. Moreover, none of the participants reported to be taking any medication.

### Ethics Statement

The study was performed in accordance with the ethical principles expressed in the Code of Ethics of the World Medical Association (Declaration of Helsinki). Paradigms and protocol employed in this study were reviewed and approved by the ethics committee of the University of Tübingen. All participants gave their written informed consent prior to inclusion in the study and received a small financial compensation for their participation.

### rTMS Procedure

The rTMS procedure comprised the determination of the motor threshold (MT) and three randomized blocks of rTMS application (one block per stimulation site) with 30-minute breaks separating the consecutive stimulation blocks.

#### Motor threshold determination

Prior to the first rTMS session, the MT was determined for the right and left motor cortex using the ‘observation of movement’ method [21]. The MT was defined as the minimum stimulation intensity required to trigger a visible twitch in the hand muscle in 5 out of 10 cases. The MT of the right motor cortex was used as reference intensity for stimulation of areas in the right hemisphere (i.e. right STC and right IFC) and the MT of the left motor cortex was used as reference intensity for stimulation of areas in the left hemisphere (i.e. left STC and left IFC). As far as the control region PZ is concerned, the mean of left and right MT was calculated and used as reference intensity.

#### rTMS parameters

Each of the two experiments consisted of three blocks of low-frequency (1 Hz) rTMS application with a duration of 14 minutes. Block order was randomized among participants. rTMS was applied offline using a MagPro X100 stimulator and a MCF-B65 figure-of-eight coil which was fixed to the scalp position using a flexible arm (MagVenture A/S, Farum, Denmark). The offline approach was chosen to avoid influences of disturbing side effects of rTMS during the acquisition of behavioral data such as muscle twitches or coil clicking. The positioning of the coil over the stimulation sites and the monitoring of the positioning throughout the stimulation was executed with help of the frameless neuronavigation system LOCALITE TMS Navigator Version 2.1.12 (LOCALITE GmbH, Sankt Augustin, Germany). To allow an individual navigation, for each participant, high-resolution T1-weighted images were acquired on a 3-T whole-body scanner using a magnetization-prepared rapid acquisition gradient echo (MPRAGE) sequence (Note: The anatomical images were acquired in four different studies with the following scanning parameters: Study 1: field of view [FoV]  = 256 mm×256 mm, 176 slices, 1 mm slice thickness, no gap, repetition time [TR]  = 2300 ms, echo time [TE]  = 4.18 ms, matrix  = 256×256, flip angle  = 9°; Study 2: FoV  = 240 mm×256 mm, 176 slices, 1 mm slice thickness, no gap, TR  = 2300 ms, TE  = 2.92 ms, matrix  = 240×256, flip angle  = 8°; Study 3: FoV  = 210 mm×210 mm, 192 slices, 1 mm slice thickness, gap 0.5 mm, TR  = 2300 ms, TE  = 2 ms, matrix  = 256×256, flip angle  = 9°; Study 4: FoV  = 256 mm×256 mm, 176 slices, 1 mm slice thickness, no gap, TR  = 2300 ms, TE  = 2.96 ms, matrix  = 256×256, flip angle  = 8°).

The exact stimulation sites were defined based on coordinates provided by a recent meta-analysis of Witteman and colleagues [13]. The original Talairach coordinates for each stimulation site as well as the respective coordinates converted to the Montreal Neurological Institute (MNI) space using the tal2mni function (http://imaging.mrc-cbu.cam.ac.uk/imaging/MniTalairach) within Matlab (MathWorks, Inc., Natick, MA, USA) are reported in the section for each experiment. The targets of stimulation were drawn around the coordinates with an accuracy of ±2 mm. A sphere of 10 mm in radius centered at the respective coordinates delineated the bounds of the target area. The control region PZ was defined for each participant by hand with the help of a measuring tape and guidelines provided by the international 10–20 system of EEG electrode placement (PZ = 20% of total distance between a participant's nasion and inion added to the location of CZ, which is half the distance between nasion and inion in the midline). The respective scalp position was then entered as a target into the program LOCALITE TMS Navigator Version 2.1.12 (LOCALITE GmbH, Sankt Augustin, Germany), and a sphere of 4 mm in radius centered around the entered target marked the bounds of the stimulation target region.

**Figure 1:**
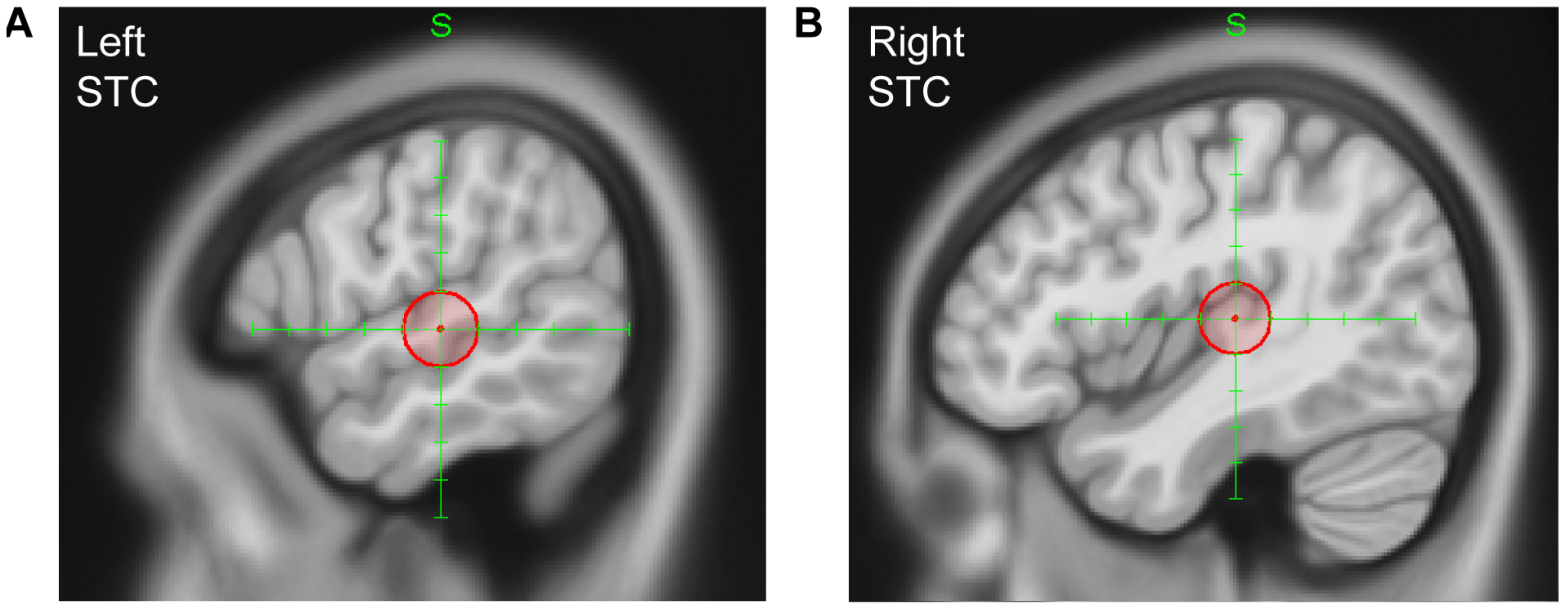
Stimulation sites in the superior temporal cortex. Location of (A) the left hemispheric and (B) the right hemispheric target area.

***Experiment 1 – rTMS of the superior temporal cortex (STC)*** ***:*** The stimulation sites were the left (Talairach: x = −58, y = −22, z = 2; MNI: x = −59, y = −23, z = 1; see Figure 1A) and right STC (Talairach: x = 44, y = −24, z = 8; MNI: x = 44, y = −25, z = 7; see Figure 1B) with PZ as a control region. Each region was stimulated separately resulting in three stimulation blocks with stimulation intensities within each block set to 120% of the MT. Depending on the individual motor thresholds, the stimulation intensities ranged between 52% and 74% (mean [*M*] = 62.77%, *SD*  = ±6.03%) of the stimulator output for the left STC, between 60% and 77% (*M* = 67.85%, *SD*  = ±5.18%) for the right STC, and between 56% and 76% (*M* = 65.38%, *SD*  = ±5.56%) for the control region PZ.

**Figure 2:**
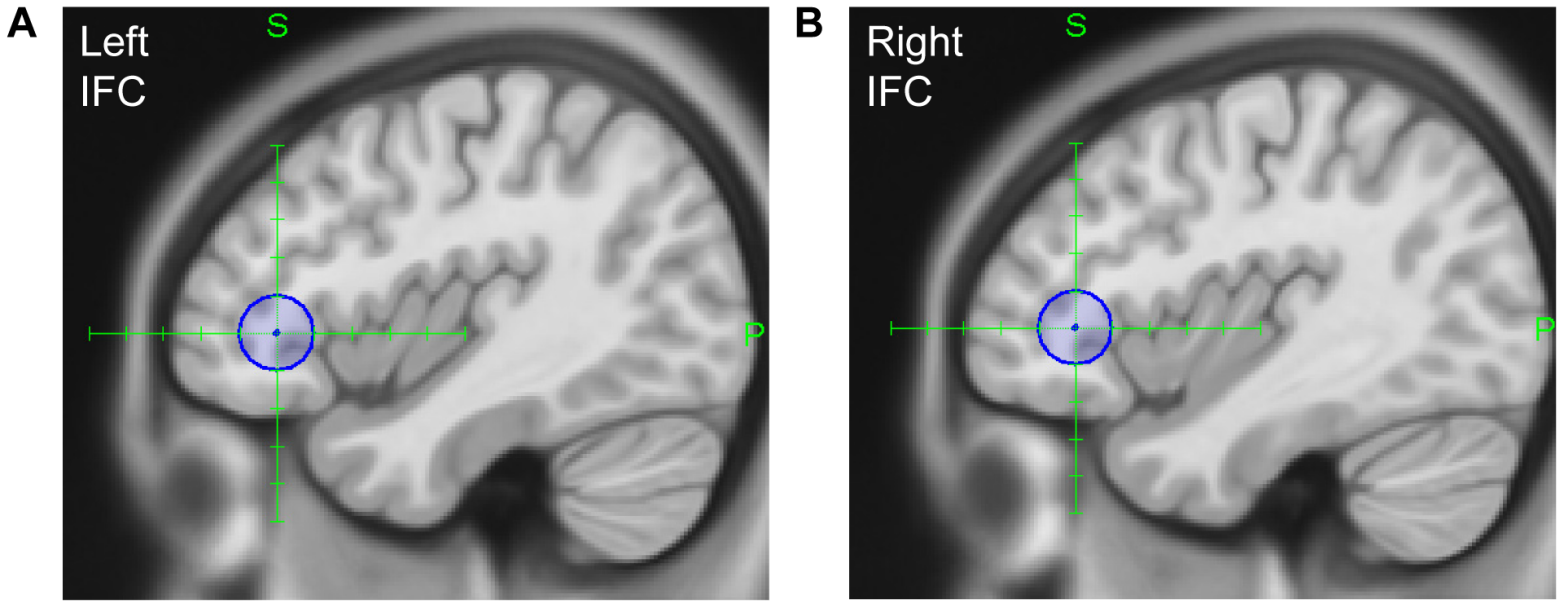
Stimulation sites in the lateral frontal cortex. Location of (A) the left hemispheric and (B) the right hemispheric target area.

***Experiment 2 – rTMS of the inferior frontal cortex (IFC)*** ***:*** The stimulation sites were the left (Talairach: x = −42, y = 34, z = 0; MNI: x = −42, y = 35, z = 2; see Figure 2A) and right IFC (Talairach: x = 40, y = 34, z = 2; MNI: x = 40, y = 35, z = 4; see Figure 2B) with PZ as a control region. Each region was stimulated separately resulting in three stimulation blocks with stimulation intensities within each block set to 100% of the MT. In contrast to Experiment 1, the stimulation intensity was lowered to 100% of the MT to avoid excessive discomfort for the participants associated with stimulation over these particular target areas. Depending on the individual motor thresholds, the stimulation intensities ranged between 40% and 66% (*M* = 52.46%, *SD*  = ±8.45%) of the stimulator output for the left IFC, between 46% and 68% (*M* = 57.23%, *SD*  = ±7.05%) for the right IFC, and between 44% and 67% (*M* = 54.85%, *SD*  = ±7.45%) for the control region PZ.

### Stimulus Material and Task

The stimuli were part of a larger stimulus set used in previous studies [22]: Four actors (2 females) spoke one of four German nouns (“Gabel” [“Fork”], “Möbel” [“Furniture”], “Objekt” [“Object”], “Zimmer” [“Room”]), rated as neutral in meaning, with either a neutral nonverbal expression or with a nonverbal expression of happiness, seduction, anger, or disgust. The stimuli had a mean duration of 838 ms (*SD*  = ±354.81 ms) and were presented in three different modalities: audiovisual (video and audio track), visual (video track only), or auditory (audio track only). In total, the stimulus material comprised a set of 60 stimuli (4 actors x 5 nonverbal expressions x 3 modalities).

Immediately after each rTMS application, all participants had to complete a task about 7 minutes in duration. Each participant watched all 60 stimuli in a randomized order. The participants' task was to evaluate the emotional state of the speaker using only the nonverbal signals. Depending on the respective modality, participants were asked to use the facial expression and tone of voice (audiovisual stimuli), only the facial expression (visual stimuli), or only the tone of voice (auditory stimuli) for their judgements. Answers were provided using a forced-choice format allowing participants to choose from one out of five categories (“neutral”, “happiness”, “seduction”, “anger”, or “disgust”). In order to avoid effects attributable to the arrangement of response categories, the arrangement of the five categories was varied among participants. Participants were asked to indicate their subjective judgement as quickly as possible via one of five buttons on a Cedrus RB-730 Response Pad (Cedrus Corporation, San Pedro, CA, USA). Answers were expected within a time frame of five seconds following stimulus onset. Participants were allowed to indicate their answers while the video or sound track was still running. Subsequent to the end of a video or sound track the five categories and the given answer were shown as feedback. The experiment was run on a standard personal computer using the software Presentation Version 16.0 (Neurobehavioral Systems Inc., Albany, CA, USA). Sound was played through loudspeakers positioned left and right of the computer monitor.

### Analysis of Behavioral Data

The hit rates and reaction times of the participants were treated as outcome variables. The data were analysed using the software package IBM SPSS Statistics Version 21 (IBM Corporation, Armonk, NY, USA). Effects of rTMS on participant's ratings were evaluated by means of a 3×3 repeated-measures analysis of variance (ANOVA) with stimulation site (left, right, control region) and modality (auditory, visual, audiovisual) defined as within-subject factors. Effects of rTMS on participants' reaction times were evaluated by means of a 3×3 ANOVA with stimulation site (left, right, control region) and modality (auditory, visual, audiovisual) defined as within-subject factors. To account for violations of sphericity, results were Greenhouse-Geisser corrected [23]. With respect to the first hypothesis guiding this research, we expected rTMS-induced disruptions of the left and right STC to be reflected in an interaction of stimulation site and modality: Stimulation over the left and right STC was expected to lead to lower hit rates and slower reaction times for stimuli of the auditory modality only. Furthermore, we assumed these effects to be stronger for rTMS-induced disruptions of the right as compared to the left STC. With respect to the second hypothesis guiding this research, we expected rTMS-induced disruptions of the left and right IFC to be reflected in a main effect of stimulation site: Stimulation over the left and right IFC was expected to lead to lower hit rates and slower reaction times for all stimuli, independent of their respective modalities.

## Results

### Hit Rates

#### Experiment 1 – rTMS of the superior temporal cortex (STC)

**Figure 3:**
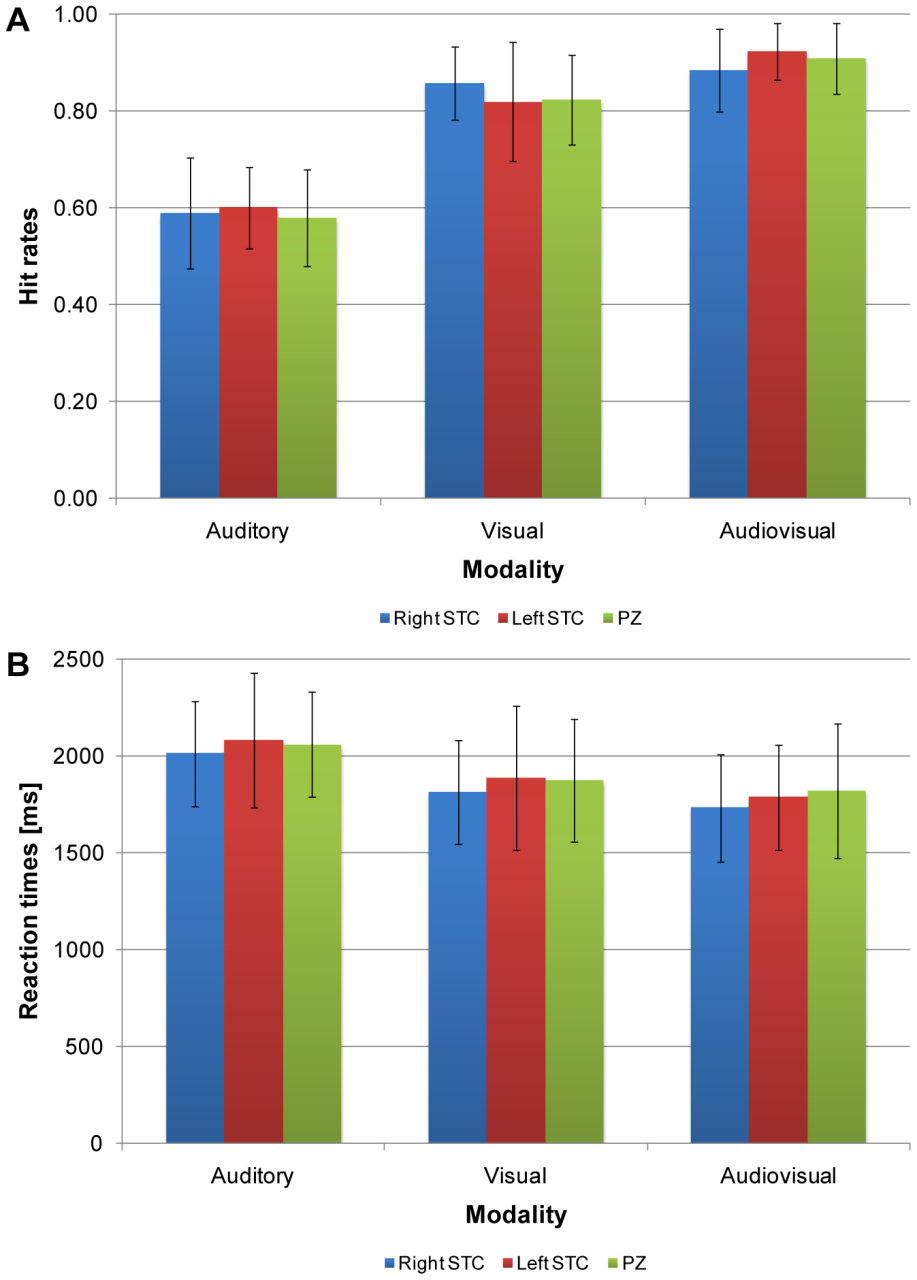
Behavioral results obtained in Experiment 1. (A) Hit rates. (B) Reaction times. Bars represent the averaged hit rates or reaction times for the three modalities in the right (blue), or left STC (red), and control region PZ (green). Error bars represent the standard error of the mean (*N* = 13).

Results indicated a main effect of modality, *F*(1.93, 23.21) = 151.70, *p*<.001, partial η^2^ = .93 (see Figure 3A). No main effect of stimulation site, *F*(1.88, 22.59) = 0.20, *p* = .81, partial η^2^ = .02, or interaction between stimulation site and modality, *F*(3.06, 36.72) = 1.02, *p* = .39, partial η^2^ = .08, was found.

#### Experiment 2 – rTMS of the inferior frontal cortex (IFC)

**Figure 4:**
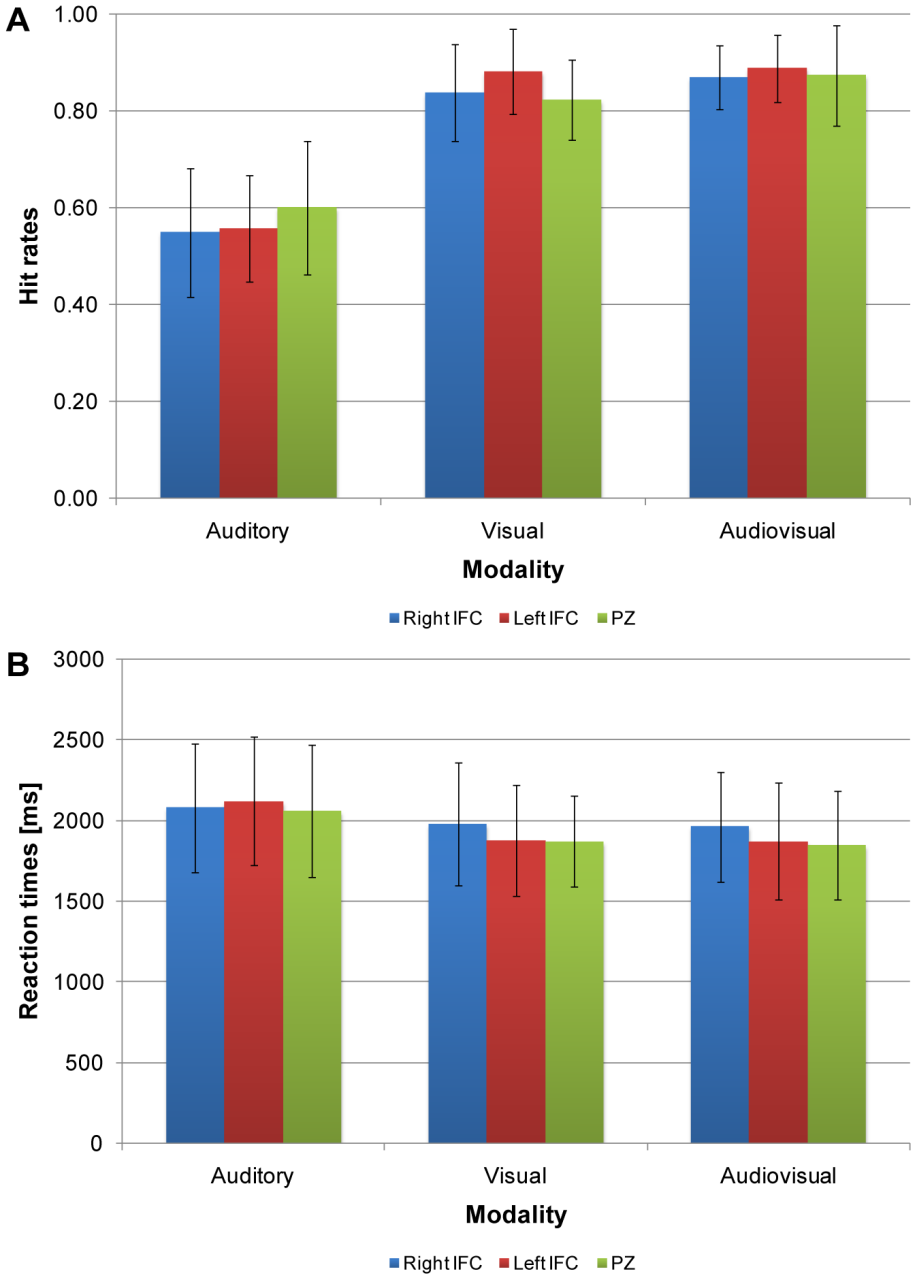
Behavioral results obtained in Experiment 2. (A) Hit rates. (B) Reaction times. Bars represent the averaged hit rates or reaction times for the three modalities in the right (blue), or left IFC (red), and control region PZ (green). Error bars represent the standard error of the mean (*N* = 13).

Results indicated a main effect of modality, *F*(1.69, 20.30) = 157.14, *p*<.001, partial η^2^ = .93 (see Figure 4A). No main effect of stimulation site, *F*(1.77, 21.28) = 0.68, *p* = .50, partial η^2^ = .05, or interaction between stimulation site and modality, *F*(3.62, 43.44) = 1.53, *p* = .22, partial η^2^ = .11, was found.

### Reaction Times

#### Experiment 1 – rTMS of the superior temporal cortex (STC)

Results indicated a main effect of modality, *F*(1.79, 21.51) = 34.82, *p*<.001, partial η^2^ = .74 (see Figure 3B). No main effect of stimulation site, *F*(1.99, 23.91) = 0.45, *p* = .64, partial η^2^ = .04, or interaction between stimulation site and modality, *F*(2.60, 31.16) = 0.23, *p* = .85, partial η^2^ = .02, was found.

#### Experiment 2 – rTMS of the inferior frontal cortex (IFC)

Results indicated a main effect of modality, *F*(1.52, 18.29) = 16.21, *p*<.001, partial η^2^ = .58 (see Figure 4B). No main effect of stimulation site, *F*(1.77, 21.23) = 0.36, *p* = .68, partial η^2^ = .03, or interaction between stimulation site and modality, *F*(2.71, 32.47) = 1.85, *p* = .16, partial η^2^ = .13, was found.

## Discussion

The goal of the present study was to evaluate and refine a current model of prosody processing [1]–[3] by investigating whether different brain structures frequently associated with different aspects of prosody perception in fact contribute to their hypothesized decoding steps. To this end, rTMS was used to study effects of reducing brain activity in prosody-related brain regions.

Regarding the model of prosody processing [1]–[3], our first hypothesis was that rTMS-induced reduction of activity in the left and right STC would interfere with the extraction and analysis of auditory information and thus should hamper the evaluation of prosodic emotional cues whereas, given the prosody-specific nature of these brain areas' contributions, no such effect should occur for visual information. However, our results did not confirm this hypothesis: No stimulation effects were found with respect to participants' hit rates or reaction times.

One might speculate that the absence of rTMS effects in our study – despite known effects from clinical data in patients with brain lesions [24]–[28] – could be due to compensatory mechanisms of the brain. For instance, it might be possible that temporary rTMS-induced activity reductions of the left or right STC were compensated by other brain structures involved in the processing of acoustic information. Such compensatory mechanisms might have prevented any measurable rTMS effects at the behavioral level. Possible “takeover candidates” could be functionally related brain regions of the same hemisphere (i.e. intrahemispheric compensation), or the homologous regions of the opposite hemisphere (i.e. interhemispheric compensation), or an interplay of the aforementioned compensatory mechanisms. This interpretive approach is in line with previous studies describing interhemispheric compensation effects, for instance, in the motor cortex [29] as well as in language-related regions [30]–[31].

Regarding the model of prosody processing [1]–[3], our second hypothesis was that rTMS effects on the left and right IFC should hamper the evaluation of nonverbal emotional stimuli, independent of their respective modalities. However, our results did not confirm this hypothesis: No main effect of stimulation site was found with respect to participants' hit rates or reaction times. As previously discussed, a possible explanation for these negative findings might be that intra- and/or interhemispheric compensation prevented any measurable rTMS effects at the behavioral level.

Interestingly, however, other studies following a similar approach to the matter showed effects of rTMS on the accuracy of voice detection [15] or the speed of prosody decoding [16]–[18]. One possible explanation for our negative findings might be that the task was too easy, thus preventing any measurable rTMS effects. To avoid such problems, for instance, Bestelmeyer and colleagues [15] adjusted the individual's performance level to approximately 80% correct before the actual TMS session. In our study, average hit rates across all three modality conditions were at a similar level (*M* = 77.00%, *SD*  = ±6.13%). It is interesting to note, however, that the hit rates differed between auditory (*M* = 57.90%, *SD*  = ±8.09%), visual (*M* = 84.01%, *SD*  = ±7.42%), and audiovisual stimuli (*M* = 89.08%, *SD*  = ±6.34%). The hit rates for auditory stimuli, thus, were even lower – indicating higher task difficulty – as compared to those reported in the study by Bestelmeyer and colleagues [15], but still no rTMS effects on the evaluation of prosodic emotional cues were found. Therefore, it seems rather unlikely that the negative findings are simply explained by a low task difficulty.

With regard to further possible explanations for our negative findings, in the following, we will focus on a comparison with two offline-low frequency studies [16], [18] due to a relatively high degree of similarity between these two studies and ours. Regarding the stimulus material, we used words and not sentences. More importantly, our stimulus material consisted of German nouns, rated as neutral in meaning. However, the latter has an impact on the degree of incongruence, which in turn may impact on rTMS effects: Combinations of neutral word meaning and emotional prosodic information lead to lower levels of information incongruency compared to combinations of emotional word meaning and emotional nonverbal cues. In comparison to weakly incongruent stimuli, strongly incongruent stimuli may have an influence on the task difficulty which in turn may play a role in determining the strength of rTMS effects in the sense that, compared to weakly incongruent stimuli, the processing of strongly incongruent stimuli may be more prone to rTMS-induced interferences. This is in line with the findings of Alba-Ferrara and colleagues [16] who showed a significant rTMS effect only for incongruent but not for congruent trials of the prosodic task. Decreases of cortical excitability associated with rTMS, thus, might have rather small effects on emotional prosody processing and may lead to misjudgments only if the stimulus comprises conflicting emotional information at verbal and nonverbal level that attract higher attention under this condition. All in all, this fits well with the assumption of intra- and/or interhemispheric compensations in the sense that the success of such compensation mechanisms might depend on the difficulty of the respective task. Relating this idea to our study, working with stimulus material including only weakly incongruent stimuli, might have restricted the possibilities to reveal rTMS effects on prosody processing. However, the study by van Rijn and colleagues [18] highlights the importance of further stimulus properties: Using combinations of neutral semantic content and emotional prosodic information, the data revealed a significant rTMS effect on reaction times, however, only for prosodic expressions of withdrawal emotions (fear and sadness), not approach emotions (anger and happiness) [18]. The latter might result from different acoustic characteristics of withdrawal vs. approach emotions [32]. Here, too, it is conceivable that the processing of some acoustic parameters is more prone to rTMS-induced interferences as compared to others.

Previous explanatory approaches notwithstanding, one should keep in mind that negative results might be due to methodological differences in the rTMS intervention. One possible explanation for our negative findings might be that the effects attained by offline low-frequency rTMS are not strong enough to influence the participants' performances. However, previous studies using this approach provide evidence to the contrary [16], [18]. More specifically, one might consider the duration of offline low-frequency rTMS as well as the duration of the task to be of importance in attaining offline low-frequency rTMS effects. To quantify these two timing parameters, we calculated their ratio. However, our ratio (14 minutes rTMS/7 minutes task  = 2) lies within the range of the ratios reported in the studies by Alba-Ferrara and colleagues (10 minutes rTMS/4 minutes task  = 2.5) [16] and van Rijn and colleagues (12 minutes rTMS/7 minutes task  = 1.7) [18]. Moreover, one might consider the sample size of critical importance. But again, our sample size (13 participants per experiment) is comparable with the sample sizes reported in the studies by Alba-Ferrara and colleagues (11 participants) [16] and van Rijn and colleagues (14 participants) [18]. As a last point we have to take into consideration that the coordinates reported in the meta-analysis of Witteman and colleagues [13] do not cover a possible inter-subject variability in location of brain regions involved in prosody processing, thus possibly preventing optimal stimulation conditions at an individual level. Therefore, in future studies, one should consider using functional localizer experiments for the selection of stimulation sites as already successfully done in the study by Bestelmeyer and colleagues [15].

In conclusion, prosody processing seems to be far more complex than it is illustrated by the existing models, calling for future research. With regard to the discussed interhemispheric compensation effects, a future study could make use of simultaneous low-frequency bilateral rTMS. However, given the discomfort associated particularly with lateral frontal stimulation, approaches employing a bilateral stimulation protocol may lead to high dropout rates and a sampling bias. Another option could be to make use of a combined rTMS-fMRI study. This method allows not only investigating low frequency rTMS-induced activity reductions but also associated compensatory activations. Moreover, since it is possible that such compensation processes are accomplished not only by the homologous region in the opposite hemisphere, but also by other regions in the same as well as in the opposite hemisphere, combining TMS and fMRI could help to identify further compensating regions. This approach could shed light on additional important brain regions involved in prosody processing, thus contributing to the extension of the existing models.
